# Intracranial Hemorrhage in a Patient With Guillain-Barré Syndrome: A Potential Complication, a Coincidental Finding or a Treatment-Related Complication

**DOI:** 10.7759/cureus.57174

**Published:** 2024-03-29

**Authors:** Sofia Kanna, George Mixides, Costas Michaelides

**Affiliations:** 1 Department of Neurology, American Medical Center, Nicosia, CYP; 2 Department of Intensive Care Unit, American Medical Center, Nicosia, CYP

**Keywords:** guillain-barre syndrome (gbs), guillain-barré, gbs variant, pharyngeal-cervical-brachial (pcb) variant, case-report, ivig use, stroke, intracranial bleeding, intracerebral hemorrhage, pharyngeal-cervical-brachial variant

## Abstract

This case report demonstrates the difficulty of diagnosing and managing the pharyngeal-cervical-brachial (PCB) variant of Guillain-Barré syndrome (GBS), as well as the rare complication of intracerebral hemorrhage (ICH). A male patient in his mid-60s, presented with bilateral upper limb weakness, bilateral ptosis, and bulbar symptoms. The clinical presentation combined with paraclinical findings supported the diagnosis of PCB. During the course of PCB, the patient required tracheostomy and gastrostomy due to the worsening of his symptoms. Eleven days after hospitalization, and six days after the course of intravenous immunoglobulin (IVIG), the patient developed intracranial bleeding. All clinicians should consider the PCB syndrome in patients with bilateral upper extremity weakness and oropharyngeal involvement, in order to develop a personalized treatment plan and closely monitor potential life-threatening complications such as ICH.

## Introduction

Guillain-Barré syndrome (GBS) is an immune-mediated peripheral polyneuropathy that is characterized by an acute-onset, symmetric muscle weakness, with decreased or absent deep tendon reflexes (DTRs) and it is usually accompanied by sensory changes and autonomic dysfunction. GBS occurs worldwide with an annual incidence rate of 1-2 cases per 100,000 people, affecting all ages, and is slightly more common in men than women, with a male-to-female ratio of 1.5:1 [1,2].

The pharyngeal-cervical-brachial (PCB) variant represents an atypical phenotype of the disease, an estimate of 3% of all cases, and constitutes a diagnostic challenge for many neurologists [3]. It is characterized by rapidly progressive oropharyngeal and cervicobrachial weakness associated with areflexia of the upper limbs and thus, the patients can be misdiagnosed as having a brainstem stroke, botulism, or myasthenia gravis.

Intracerebral hemorrhage during the course of GBS is an extremely rare and potentially fatal complication. The exact pathophysiology remains unknown.

## Case presentation

**Figure 1:**
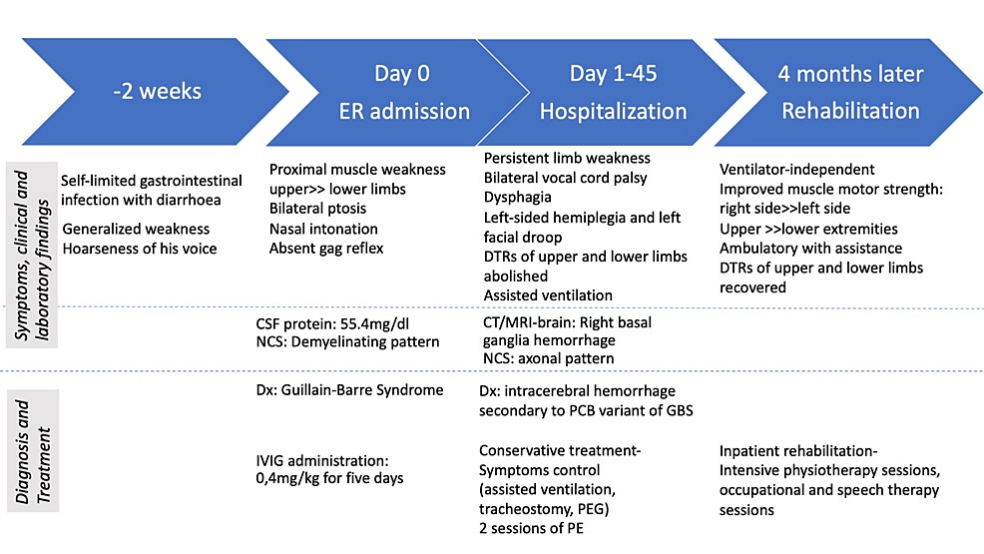
Case report timeline CSF: Cerebrospinal fluid, NCS: Nerve conduction studies, Dx: Diagnosis, IVIG: Intravenous immunoglobulin, DTRs: Deep tendon reflexes, PCB: Pharyngeal-cervical-brachial, GBS: Guillain-Barré syndrome, PEG: Percutaneous endoscopic gastrostomy, PE: Plasma exchange

A 65-year-old male patient with a past medical history of type II diabetes mellitus was admitted to our institution with a nine-day history of generalized weakness and hoarseness of voice. The case timeline is shown in Figure 1. He reported diarrhea 12 days prior to the onset of symptoms. On admission, he was hemodynamically stable, with a Glasgow Coma Scale (GCS) of 15/15. His oxygen saturation was 97%. Neurological examination was positive for generalized weakness with the Medical Research Council’s (MRC) scale of muscle strength grade 4/5 proximally on upper extremities, and 5-/5 proximally on lower extremities. There was bilateral ptosis, without ophthalmoplegia. The patient had absent gag reflex and nasal intonation, suggestive of bulbar involvement. The DTRs were present in the lower extremities (2+) more than in the upper extremities (1+) and he had flexor plantar responses. The patient was admitted to the ICU given the suspicion for GBS for close monitoring of the airway. A nasogastric tube was inserted.

A lumbar puncture was performed on admission. Cerebrospinal fluid analysis revealed a normal cell count and glucose levels, but the protein levels were elevated to 55.4 mg/dL - (range: 15.0-45.0mg/dL) - thus revealing albuminocytologic dissociation.

Brain MRI was normal, and whole spine MRI showed enhancement of the nerve roots of the conus medullaris. There was also performed a CT angiography of the cerebral arteries, on admission, without significant findings.

The nerve conduction studies (NCS) on admission were supportive of the presence of acute demyelinating neuropathy, as shown in Table 1.

**Table 1:** The nerve conduction studies (NCS) on admission were supportive of acute demyelinating neuropathy. The NCS revealed a conduction block of the right ulnar nerve over the elbow groove, prolonged and unobtainable F-waves of the right median and ulnar nerves respectively, and decreased right tibial nerve conduction velocity, suggesting the presence of acute demyelinating neuropathy. NP: negative peak, PP: positive peak, R: right, L: left, APB: abductor pollicis brevis, ADM: abductor digiti minimi, EDB: extensor digitorum brevis, AH: abductor hallucis, FVC: forced vital capacity, GBS: Guillain-Barré syndrome

Sensory Nerve Conduction Study										
Nerve/Sites	Rec. Site	Onset Lat (ms)	Peak Lat (ms)			NP Amp (µV)	PP Amp (µV)	Segments	Distance (mm)	Velocity (m/s)
R Median - Dig II (Antidromic)										
Wrist	Index	3.18	4.01			18.6	37.9	Wrist - Index	140	44
R Ulnar - Dig V (Antidromic)										
Wrist	Dig V	2.29	3.02			13.1	28.7	Wrist - Dig V	140	61
Motor Nerve Conduction Study										
Nerve/Sites	Muscle	Latency (ms)		Amplitude (mV)		Segments	Dist. (mm)	Lat Diff (ms)	Velocity (m/s)	
R Median - APB										
Wrist	APB	4.19		4.9		Wrist - APB	80			
Elbow	APB	9.27		3.3		Elbow - Wrist	250	5.08	49.2	
R Ulnar - ADM										
Wrist	ADM	3.85		1.3		Wrist - ADM	80			
B.Elbow	ADM	8.25		1.3		B.Elbow - Wrist	240	4.40	54.6	
A.Elbow	ADM	10.54		0.5		A.Elbow - B.Elbow	90	2.29	39.3	
R Peroneal - EDB										
Ankle	EDB	4.42		2.7		Ankle - EDB	80			
B. Fib Head	EDB	12.88		2.1		B. Fib Head - Ankle	340	8.46	40.2	
A. Fib Head	EDB	14.46		1.7		A. Fib Head - B. Fib Head	90	1.58	56.8	
R Tibial - AH										
Ankle	AH	5.31		3.9		Ankle - AH	80			
Knee	AH	18.46		2.4		Knee - Ankle	380	13.15	28.9	
F Waves										
Nerve	F Latency (ms)	M Latency (ms)			F-M Lat (ms)					
R Tibial - AH	27.5	5.6			21.9					
R Peroneal - EDB		46.8								
R Median - APB	33.9	4.3			29.5					

Repetitive nerve stimulation was performed to investigate the possibility of neuromuscular junction dysfunction and was found to be normal. Flexible laryngoscopy showed bilateral vocal cord paralysis and absent gag reflex.

His anti-ganglioside antibodies (anti-GD1a) were positive and the anti-GT1a antibodies were equivocal. Meanwhile, the remaining ganglioside antibodies were negative. The rest of the autoimmune screen, including anti-nuclear, anti-mitochondrial, and anti-smooth muscle antibodies, yielded negative results. Acetylcholine receptor antibodies and muscle-specific receptor tyrosine kinase (anti-MUSK) antibodies were also negative and this, along with the normal repetitive nerve stimulation study, led to the exclusion of myasthenia gravis as a possible diagnosis. *Campylobacter jejuni* serology was negative, despite the history of diarrheal illness. Serologic assay for Epstein-Barr virus viral capsid antigen immunoglobulin G (EBV VCA IgG) antibodies was positive with extremely high titers (>50IU).

A working diagnosis of GBS, PCB variant was made and the administration of intravenous immunoglobulin (IVIG) was started at a daily dose of 0.4g/kg for five days, a day after his admission. However, no clinical improvement was observed in the days following the completion of the course. Furthermore, his DTRs were abolished in all extremities. The muscle weakness remained more severe in the proximal upper extremities than in the lower extremities.

**Figure 2:**
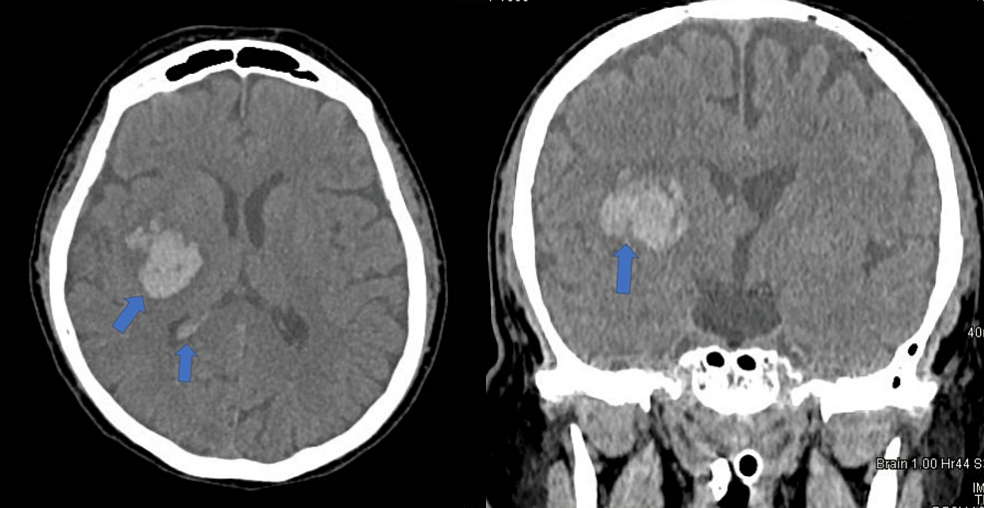
(A) Axial brain CT revealing right basal ganglia intracerebral hemorrhage with intraventricular extension. (B) Coronal CT brain of the same patient revealing right parenchymal hemorrhage with moderate midline shift.

Six days after the completion of IVIg and 11 days after admission, the patient developed left-sided hemiplegia and left facial droop. CT of the brain revealed a spontaneous hemorrhage in the right basal ganglia with intraventricular involvement and moderate midline shift, as shown in Figure 2. The patient remained hemodynamically stable, throughout his hospitalization with blood pressure (BP) monitored every hour. BP levels were maintained below 140/80 mm Hg leading up to the day of the hemorrhage and maintained stable during the days after. No further clinical indications of autonomic dysfunction were observed such as acute heart arrhythmias, diarrhea, or urine retention. Platelet count and coagulation parameters stayed within normal limits. Therefore, we were not able to repeat CT angiography of the cerebral vessels, at the time of the bleed, to detect any possible vasoconstriction. The hemorrhage was reviewed by neurosurgery who recommended immediate conservative management. This included maintaining systolic BP levels within 140-160mm Hg, intracranial pressure <20mm H_2_0, normothermia, normoglycaemia, normocarbia, euvolemia, sodium levels within 140-150mmol/L and positioning of the head at 30^o^ elevation.

**Figure 3:**
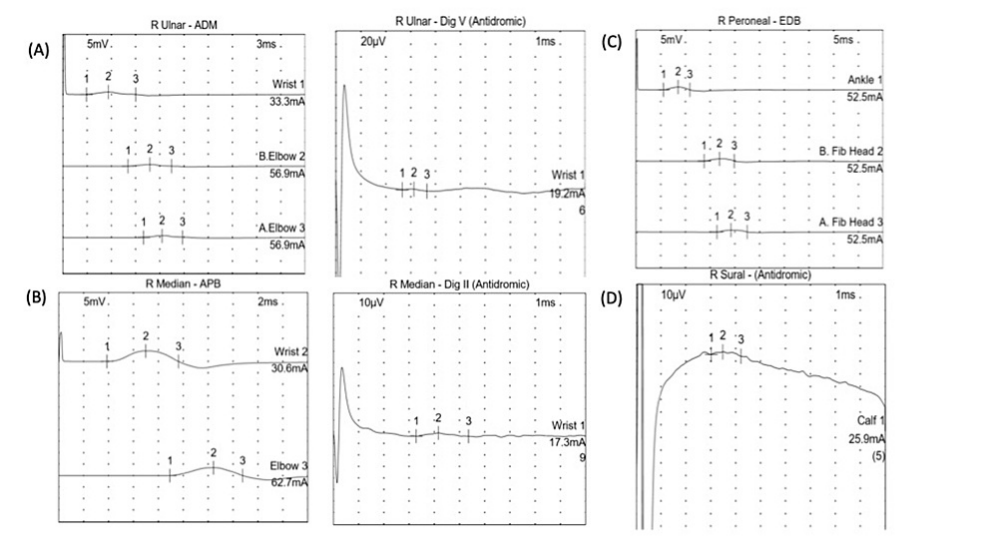
Nerve conduction studies showing evolution into an axonal pattern (A) right ulnar nerve motor conduction and sensory test; (B) right median nerve motor conduction and sensory test; (C) right peroneal nerve motor conduction test; (D) right sural nerve sensory test. APB: abductor pollicis brevis, ADM: abductor digiti minimi, EDB: extensor digitorum brevis, R: right

Repeat NCS were performed, 10 days after the first NCS, and showed an overall deterioration and evolution into an axonal pattern, with decreased amplitudes of all nerves tested and with no improvement of conduction velocities or distal latencies. The findings are shown in Figure 3.

The motor strength on the right side became 3/5, due to ongoing GBS, while the left side remained plegic, as a result of the ICH. (MRC scale: right upper extremity (RUE) proximal 2/5-distal 4/5, right lower extremity (RLE): proximal 4/5-distal 4/5). The patient’s airway control deteriorated due to the impairment of the cough reflex. The patient underwent serial spirometry (expiratory vital capacity (EVC)) on admission which was 80% of the predicted value and scored 11/32 on the modified Erasmus GBS Respiratory Insufficiency Score (mEGRIS) indicating a probability of less than 10% for mechanical ventilation within seven days of admission. However, the patient required assisted ventilation within that timeframe. Due to his clinical deterioration seven days after completion of the IVIg course, five sessions of plasmapheresis, on a total volume of 15L, were performed. The patient remained hemodynamically stable during the procedure and no noticeable side effects, such as hypotension, were observed. However, his weakness did not improve, and four days later the patient required a tracheostomy for secretion control. In the following days, another five sessions of plasmapheresis were performed. Repeated otolaryngologist assessment showed no improvement of the vocal cord paralysis and absence of gag reflex, therefore a percutaneous endoscopic gastrostomy (PEG) was placed 39 days after admission. Over the following several days the patient showed a gradual improvement of respiratory function and became ventilator-independent. The patient was referred for inpatient rehabilitation services including intensive physiotherapy, speech therapy, and occupational therapy sessions.

Four months after onset, the patient had a tracheostomy for secretion management and his neurological examination improved gradually following intensive rehabilitation. His cognitive function remained normal. There was mild left facial weakness, while the bilateral vocal cord palsy and dysphagia persisted. The muscle motor strength in the RUE was 4-/5 proximally and 5-/5 distally. The left upper extremity (LUE) improved (2/5). He became ambulatory with assistance with near normal strength in his RLE and improvement of his left lower extremity (LLE) at 3/5. The DTRs of both upper and lower limbs recovered.

## Discussion

PCB forms a localized type of axonal GBS that is characterized by bulbar involvement and upper limb weakness, in the absence of ophthalmoplegia and ataxia. The mean age of onset is 43 years old and appears to be slightly more prominent in men [3]. Our patient met the proposed diagnostic criteria of the PCB variant according to Table 2, after the exclusion of other treatable causes [3].

**Table 2:** Diagnostic criteria for the pharyngeal-cervical-brachial (PCB) variant of Guillain-Barré syndrome (GBS) Source: Reference [3]

Features required for diagnosis	Features strongly supportive of the diagnosis
1. Relatively symmetric oropharyngeal weakness AND neck weakness AND arm weakness AND arm areflexia/ hyporeflexia	1. Antecedent infectious symptoms
2. Absence of ataxia AND disturbed consciousness AND prominent leg weakness	2. Cerebrospinal fluid albuminocytological dissociation
3. Monophasic illness pattern AND interval between onset and nadir of oropharyngeal or arm weakness between 12 h and 28 days AND subsequent clinical plateau	3. Neurophysiological evidence of neuropathy
4. Absence of identified alternative diagnosis	4. Presence of IgG anti-GT1a or anti-GQ1b antibodies

Six days after the initial treatment of IVIG, the patient developed a right basal ganglia hemorrhage with intraventricular involvement, as shown in Figures 2a-2b. This is an extremely rare complication of GBS but has been reported [4-7]. Possible underlying mechanisms for the development of ICH in GBS have been postulated and involved both the use of immunoglobulins, but also the the condition itself.

A small number of patients with GBS developing cerebral bleeding shortly (usually 2 to 10 days [4]) after receiving IVIG treatment have been reported in the literature [5-7]. Immunoglobulin use has been associated with both ischemic and hemorrhagic strokes, with the first ones being more common [4]. The postulated mechanisms include IVIG-related hemodynamic changes affecting BP levels (hypotension or hypertension) and increasing cardiac rate [4]. However, these changes were not recorded in our patient. Additional presumptive mechanisms are intravascular hypercoagulopathy, autoimmune complications such as vasculitis, hyperviscosity, and vasospasm of the brain vessels. Reversible cerebral vasoconstriction syndrome (RCVS) can also lead to both ischemic and hemorrhagic strokes and can be diagnosed with CT angiography at the time of the event [4].

Nevertheless, GBS itself may play an important role in the pathogenesis of intracranial hemorrhage. More specifically, severe hypertension due to autonomic neuropathy can cause a dysfunction of the cerebral autoregulation mechanism and rupture of cerebral vessels [4,8]. However, there is limited information available about the exact pathophysiology of the ICH in GBS patients, and thus, further research is needed.

The management of PCB patients requires both immunotherapy and symptomatic treatment [9]. Our patient received treatment with IVIG, as well as, plasmapheresis sessions, and improved gradually in terms of his strength and mobility. However, the vocal cord paralysis persisted without any indication of improvement, rendering him dependent on long-term tracheostomy and gastrostomy. Previous reports suggest that bulbar involvement is associated with positive anti-Gt1a antibodies [9]. However, the titers of these antibodies in our case were equivocal. Interestingly, he exhibited bilateral ptosis without ophthalmoplegia, indicating the involvement of multiple cranial nerves. This is not a common manifestation of PCB syndrome, nonetheless, it has occasionally been observed [3].

The prognosis of GBS and PCB subtypes varies because of the different clinical manifestations of the disease, with the majority of the patients recovering completely within a year [9]. However, more than one in five patients with GBS may have a residual deficit, and an estimated 5% of patients will die [10]. The main factors associated with poor outcomes are age over 40, antecedent diarrheal illness, and high disability at the onset of the disease [10]. Physiotherapy, occupational therapy, speech and language therapy can enhance the patient’s quality of life [9]. Thus, personalized treatments are needed to improve the outcome of each patient [10]. When GBS is complicated by cerebral bleeding, the prognosis worsens even more. Taking into consideration the existing data, most of the reported patients showed only limited recovery [11].

The strengths of our case include the unusual presentation of GBS, which can be mistaken for botulism, myasthenia gravis, or a brainstem stroke. Botulism was excluded on the basis of CSF findings, normal pupil function, and the absence of ophthalmoplegia, while myasthenia gravis was also excluded after serological and electrophysiological evaluation. Brainstem stroke was also ruled out after normal brain imaging findings. Additionally, the presence of a potential association of ICH with either the GBS itself, or the use of immunoglobulin is supported. In fact, in the absence of underlying risk factors for ICH in our patient, we cannot ignore the possibility that this could have been a GBS-related complication or secondary to IVIG use.

A few limitations of this study should be acknowledged. The exact mechanism of the cerebral bleeding could not be confirmed. We were not able to perform a CT angiography of the cerebral vessels at the time of the bleed and therefore we cannot exclude the vasoconstriction as a cause of the bleed. Furthermore, the sample of reported cases is limited making it challenging to draw a safe conclusion as to whether the ICH in a GBS patient represents a potential complication, a coincidental finding, or a complication of treatment.

## Conclusions

The PCB variant has been characterized as a rare, local subtype of GBS, and represents a diagnostic challenge. However, the association between GBS and ICH needs to be explored further due to multiple factors interacting.

Our report demonstrates that in any patient with GBS who develops new neurological findings, ICH should be included in the differential diagnosis. All neurologists should be aware both of the PCB variant of GBS and also this potentially life-threatening complication. Early diagnosis, prolonged follow-up, and supportive care are fundamental for a better prognosis for these patients.
